# The diagnostic accuracy and prognostic value of OCT for the evaluation of the visual function in children with a brain tumour: A systematic review

**DOI:** 10.1371/journal.pone.0261631

**Published:** 2021-12-23

**Authors:** Myrthe A. Nuijts, Saskia M. Imhof, Nienke Veldhuis, Coco C. Dekkers, Antoinette Y. N. Schouten – van Meeteren, Inge Stegeman

**Affiliations:** 1 Department of Ophthalmology, University Medical Centre Utrecht, Utrecht, The Netherlands; 2 Faculty of Medicine, Utrecht University, Utrecht, The Netherlands; 3 Department of Neuro-Oncology, Princess Máxima Centre for Paediatric Oncology, Utrecht, The Netherlands; 4 Department of Otorhinolaryngology and Head & Neck Surgery University, University Medical Centre Utrecht, Utrecht, The Netherlands; 5 Brain Centre Rudolf Magnus, University Medical Centre Utrecht, Utrecht, The Netherlands; 6 Epidemiology and Data Science, Amsterdam University Medical Centre, University of Amsterdam, Amsterdam, The Netherlands; Transilvania University of Brasov: Universitatea Transilvania din Brasov, ROMANIA

## Abstract

**Purpose:**

To systematically review the evidence on the diagnostic accuracy and prognostic value of retinal optical coherence tomography (OCT) to detect visual acuity (VA) or visual field (VF) loss in children with a brain tumour.

**Methods:**

PubMed, Embase and Cochrane Library databases were searched from inception to February 2021. We included studies evaluating retinal OCT and standard visual function parameters (VA and or VF) in children with a brain tumour. Two authors independently extracted data from each included study. They also assessed the methodological quality of the studies using the QUADAS-2 or QUIPS tool. The diagnostic accuracy of OCT was evaluated with receiver operating characteristic analysis, sensitivity, specificity, positive predictive value and negative predictive value. The prognostic value of OCT was evaluated with predictive measures (odds ratio).

**Results:**

We included five diagnostic studies, with a total of 186 patients, all diagnosed with optic pathway glioma. No prognostic studies were eligible for inclusion. Included studies evaluated either retinal nerve fiber layer (RNFL) thickness or ganglion cell layer—inner plexiform layer (GCL-IPL) thickness. There was considerable heterogeneity between OCT devices, OCT protocols, visual function parameters and threshold values. Sensitivity and specificity for RNFL thickness measurement ranged from 60.0% to 100.0% and 76.6% to 100%, respectively. For GCL-IPL thickness measurement, area under the curve ranged from 0.91 to 0.98 for different diameters.

**Conclusion:**

The literature regarding the diagnostic accuracy and prognostic value of OCT parameters in children with a brain tumour is scarce. Due to heterogeneity and a considerable risk of bias of included studies, we cannot draw solid conclusions regarding the accuracy of retinal OCT. Future research should investigate the potential of OCT as diagnostic and prognostic tool for the evaluation of the visual function and detection of visual impairment in children with any type of brain tumour.

## Introduction

Optical coherence tomography (OCT) is a non-invasive, in vivo, imaging modality which provides high-resolution cross-sectional images of ocular tissues by using low-coherence interferometry [1, 2]. The high resolution of modern OCT images enables the clinician to easily distinguish between multiple retinal layers around the optic nerve head and the macula. Numerous clinicians have used OCT to measure the peripapillary retinal nerve fiber layer (RNFL) thickness and the ganglion cell layer-inner plexiform layer (GCL-IPL) thickness as a surrogate marker for optic nerve swelling and or retinal ganglion cell damage [3–5]. In adults with compressive optic neuropathies or glaucoma, OCT was shown to detect a decrease in RNFL and GCL-IPL thickness, which correlates with a decline of visual function (i.e. visual field (VF) defects) [6, 7]. Furthermore, the high intervisit reproducibility of OCT measurements validates its utility for the follow-up of these patients [6–8].

In recent years, there has been increasing information about the diagnostic and prognostic ability of RNFL and GCL-IPL thickness measurements for the detection of visual acuity (VA) and VF loss in children with a brain tumour. This applies particularly to children with a brain tumour located along the visual pathway, including low-grade gliomas, craniopharyngiomas and germ cell tumours [3, 9–15]. An impaired visual function often has important long-term implications for the development, quality of life and later prospects in childhood brain tumour survivors [16, 17]. Therefore, early detection of impaired visual function and timely initiation of treatment or referral for visual rehabilitation are important to preserve visual function and improve coping in daily life [18, 19].

Regrettably, ophthalmological examination for the objective measurement of disease progression and evaluation of the visual function is challenging in children with a brain tumour. Formal VA and VF testing has limitations because these testing methods need full cooperation and cognitive ability of the patient [18, 20, 21]. Also, previous studies showed that 2D tumour volume changes on magnetic resonance imaging (MRI) do not relate to VA or VF loss [22, 23]. In these children, OCT measurements may be helpful to provide indirect information about the child’s visual status and assist in treatment decisions by the ophthalmologist and neuro-oncologist. The application of OCT has been limited in children because the traditional table-mounted OCT device requires the child’s ability to fixate and cooperate. However, with the incorporation of eye tracking technology and the development of a handheld OCT (HH-OCT) device this technique can now be successfully used in the paediatric population as well, even in very young children under general anaesthesia [3, 24–29]. In this study, we systematically review the diagnostic accuracy and prognostic value of retinal OCT to detect VA or VF loss in children with any type of brain tumour.

## Methods

### Protocol and registration

This systematic review was registered in the international prospective register of systematic reviews (PROSPERO) on April 11, 2019 (ID: 125785). Results were reported according to the principles of the Preferred Reporting Items of Systematic Reviews and Meta-Analyses (PRISMA) statement [30]. In accordance to Dutch guidelines, no institutional ethical review board approval was required.

### Information sources and search strategy

We conducted a systematic search in the Cochrane Library, Embase and PubMed on February 2, 2021. The electronic databases were searched for a combination of the following key search terms and or their synonyms: ‘glioblastoma’, ‘optic pathway glioma (OPG)’, ‘astrocytoma’, ‘craniopharyngioma’, ‘germ cell tumor’, ‘pineal tumor’, ‘medulloblastoma’, ‘ependymoma’, ‘atypical teratoid rhabdoid tumor’, ‘diffuse intrinsic pontine glioma’, ‘choroid plexus tumor’, ‘primitive neuroectodermal tumor’, ‘brain tumor’, ‘visual pathway’, ‘chiasm compression’ and ‘optical coherence tomography’. The full search strategies are presented in S1 File. There were no date or publication restrictions. We manually searched the reference lists of the included studies to ensure that no relevant studies were missed by our search strategy. No language restrictions were applied. No trial registries were sought for unpublished trials and study authors were not contacted to identify additional studies.

### Study selection

Two reviewers (M.N. and N.V.) independently screened titles and abstracts of studies identified from the electronic searches using Rayyan QCRI [31]. Full text articles of definitely or potentially relevant abstracts were obtained and reviewed for eligibility by the same two reviewers. Discrepancies were resolved by consensus or by discussion with a third reviewer (I.S.). The reviewers were unmasked for article authors, journal, institution and study results during the assessment.

### Eligibility criteria

We included studies of all designs that produce estimates of test accuracy and prognostic factor measurement or provide sufficient data from which these estimates can be computed: cross-sectional, longitudinal, cohort and case-control studies. To evaluate diagnostic accuracy, we included studies with VA and VF as reference standard and retinal OCT parameters (RNFL and GCL-IPL thickness) as index test. Studies including only patients older than 18 years of age, studies including only patients without a brain tumour, case reports including < 2 patients and studies lacking data on VA, VF and OCT parameters were excluded. In addition, studies providing insufficient data to construct 2x2 tables to estimate sensitivity (SN), specificity (SP), positive predictive value (PPV) and negative predictive value (NPV) were excluded.

### Outcome measures

The primary outcome measures of this systematic review were retinal OCT parameters (RNFL and GCL-IPL thickness measurements), VA and VF. The diagnostic accuracy of OCT was evaluated with SN, SP, PPV, NPV and receiver operating characteristic (ROC) analysis. The prognostic value was assessed with predictive measures (odds ratio). If these numbers were not reported by the authors, we calculated them with the available data.

### Assessment of methodological quality

Risk of bias and applicability concerns were assessed by two reviewers (M.N. and N.V.) independently, using the Quality Assessment of Diagnostic Accuracy Studies (QUADAS-2) tool [32] and the Quality In Prognosis Studies (QUIPS) tool [33]. Any disagreements between the reviewers were resolved by consensus.

The QUADAS-2 tool was designed to assess the methodological quality of primary diagnostic accuracy studies and facilitates assessment across four domains: patient selection, index test, reference standard and flow and timing. Each domain was assessed in terms of risk of bias and the first three domains were also assessed in terms of applicability concerns. The risk of bias within each domain was based on signalling questions and was expressed as high (+), low (-) or unclear (?). Risk of bias was rated as high if one or more items were answered with ‘no’, as low if all items were answered with ‘yes’ and as ‘unclear’ in all other instances. The definitions used for assessing the methodological quality of diagnostic accuracy studies with the QUADAS-2 tool are shown in S1 Table.

The QUIPS tool was developed for evaluating the methodological quality of prognostic studies. The following six domains were assessed: study participation, study attrition, prognostic factor measurement, outcome management, study confounding and statistical analysis and reporting. Each domain consisted of multiple items that were judged separately. The risk of bias within each domain was based on the ratings of these items and was expressed as high (+), low (-) or unsure (?). Risk of bias was rated as high if one or more items were answered with ‘no’, as low if all items were answered with ‘yes’ and as ‘moderate’ in all other instances. The definitions used for assessing the methodological quality of prognostic studies with the QUIPS tool are shown in S2 Table.

### Data analysis and synthesis

All data were extracted independently by two review authors (M.N. and N.V.). A standardized data extraction form was used, including the following items: author, country, study design, study size, gender and age of patients, type of brain tumour, presence of NF1, type of ophthalmological testing methods, type of OCT device and protocol, follow-up period and ophthalmological outcome measures. Authors of the eligible primary studies were contacted to obtain additional study data if there was insufficient data for study inclusion. We quantified the extracted data per item and presented numbers for each item in different tables.

## Results

### Results of the search

**Fig 1:**
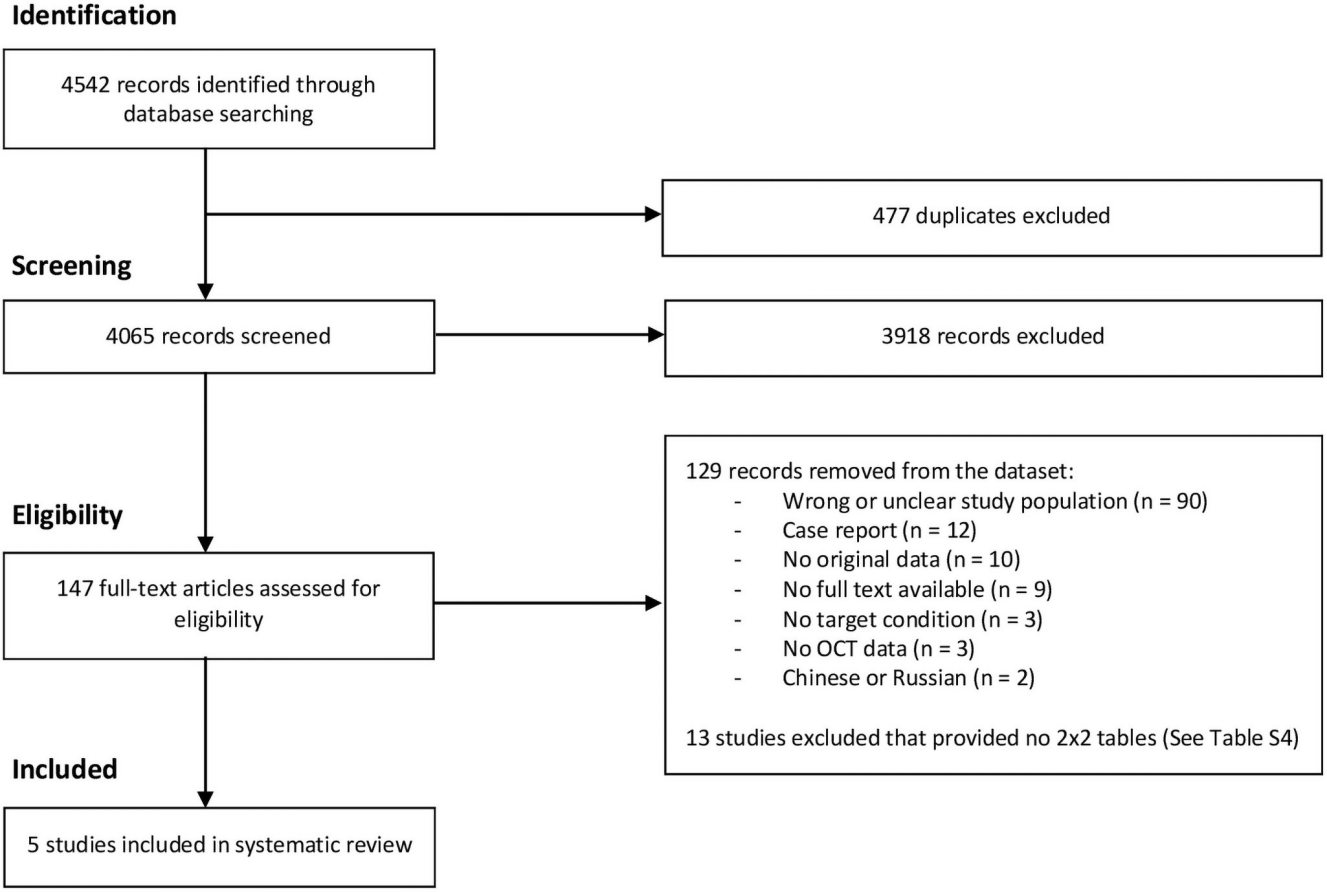
PRISMA flow chart for identification and selection of studies. OCT: optical coherence tomography.

We identified 4542 records through our literature search. After deduplication and assessment of title and abstracts, we assessed 147 records via full-text screening. Of these, we removed 129 records that included the wrong or an unclear study population (N = 93), did not contain original data (N = 10), did not contain OCT data (N = 3), case reports including <2 patients (N = 12) and records of which the full text was not available (N = 9) or that were written in Chinese or Russian (N = 2). Of the remaining 18 studies, 13 studies including both patients with and without the target condition were excluded, because they provided insufficient data to construct 2x2 tables to estimate SN, SP, PPV and NPV (S3 Table). The remaining five studies were included in this review. No additional studies were included after reference screening. A detailed overview of the identification and selection process for included studies and reasons for exclusion after full-text screening is shown in Fig 1.

### Study and patient characteristics

We found no studies evaluating the prognostic value of OCT. All five included studies assessed the diagnostic accuracy of OCT (Table 1). The studies were prospectively conducted, two were longitudinal cohort studies [12, 13] and three were cross-sectional studies [34–36]. Studies were published in English between 2013 and 2018 and were conducted in the United States of America [13, 35, 36], Iran [12] and Italy [34].

**Table 1:** Characteristics of included studies and their patient population.

First author (year)	Study design	Diagnostic or prognostic	Country	No. of patients/no. of eyes	No. of patients with NF1	Gender (M/F)	Mean age (years)	Tumour type	Follow-up time (months)
Avery (2014) [35]	Cross-sectional case-control study	Diagnostic	USA	53/95	25	28/25	OPG: median 4.8 (1.8–12.6)	OPG	NA
				OPG: 33/64					
				Control group: 20/31			Control group: median 8.7 (1.7–16.7)		
Avery (2015) [13]	Longitudinal cohort study	Diagnostic	USA	46/55	31 eyes	20/35 eyes	Stable vision: 6.5 (1.2–17.1)	OPG	New vision loss: mean 16.5 (6.1–34.1);
				Stable vision 39/45	Stable vision: 29 eyes				
				New vision loss: 7/10	New vision loss: 2 eyes		New vision loss: 6.9 (1.1–17.8)		Stable vision: 13.4 (5.7–23.7)
Fard (2013) [12]	Longitudinal cohort study	Diagnostic	Iran	23/38	6	11/12	5.8 (4–9.3)	OPG	24
Gu (2014) [36]	Cross-sectional cohort study	Diagnostic	USA	26/47	20	15/15	Normal vision: 5.3 (2.5–12.6)	OPG	NA
				Normal vision: 19/31	Normal vision: 16/19	Normal vision: 9/10			
				Abnormal vision: 11/16	Abnormal vision: 4/11	Abnormal vision: 6/5	Abnormal vision: 6.1 (2.6–12.8)		
Parrozzani (2018) [34]	Cross-sectional cohort study	Diagnostic	Italy	38/66	38	19/19	7.6±3.6	OPG	NA

NA: Not applicable; NF1: Neurofibromatosis type 1; OPG: Optic pathway glioma; USA: United States of America.

The included studies comprised in total 186 children (301 eyes) and study sample sizes ranged from 23 to 53 children (mean of 37 children [60 eyes]). With regard to the included children, 73 children were males [12, 34–36] and one study reported the inclusion of 20 male eyes and 35 female eyes [13]. Mean age ranged from 5.3 to 7.6 years [12, 13, 34, 36] and median age ranged from 4.8 to 8.7 years [35]. All children were diagnosed with an OPG (sporadic or NF1 related). The included studies investigated whether there was a relation between the visual function (VA, VF) and structural changes (OCT parameters).

### Visual outcome measures and definitions of included studies

The cut-off values for VA and VF loss varied between the studies. Two studies considered a loss of ≥ 0.2 logMAR compared to the baseline visit as significant decline in VA [12, 13], two studies defined abnormal VA as ≥ 0.2 logMAR below age-based norms [35, 36] and one study considered an abnormal VA as any VA below age-based norms [34]. With regard to VF, one study considered progressive VF loss as 3 or more contiguous points reaching significance (*P* < 0.05) using Humphrey 24–2 or as any constriction greater than 10 degrees across a minimum of 3 contiguous 15-degree vectors using the V-4-E or I-4-E isopter on Goldmann kinetic perimetry [13], one study defined progressive VF loss if mean VF mean deviation worsened by 5 dB or more [12], two studies predefined an abnormal VF as a VF defect in any quadrant [35, 36] and in one study the definition for abnormal VF was unclear [34].

Type of OCT device differed between the included studies (Table 2). Two studies used handheld SD- OCT (HH-OCT)(Bioptigen) [35, 36], two studies used tabletop SD-OCT (Spectralis, Heidelberg Engineering) [12, 34] and one study used HH-OCT (Bioptigen) as well as tabletop SD-OCT (Spectralis, Heidelberg Engineering) [13]. A detailed overview of the used OCT protocols is presented in Table 2. With regard to the three cross-sectional studies, abnormal RNFL and GCL-IPL thickness was determined as the lower fifth and first percentile in the normal-vision OPG group in two studies [35, 36] and one study calculated a ROC curve to determine the cut-off value of the RNFL thickness between the normal VA group and the abnormal VA group [34]. In the two longitudinal studies, change of RNFL thickness was defined as a decline of ≥ 10% in global RNFL thickness compared to the baseline visit [13] or as a decline of > 5 μm in average RNFL thickness compared to the previous visit [12].

**Table 2:** Visual function and OCT parameters as reported in included studies.

**First author (year)**	**OCT device**	**OCT protocol**	**Comparison** (no. of patients/ no. of eyes)	**Visual function parameters**	**OCT parameters**	**Baseline VA**	**Baseline VF**
			Avery (2014) [35]					HH-OCT (Bioptigen)	6x6 mm rectangular scan centered on the optic nerve head using 1000 A-scans across 100 B-scans.	Normal vision (49 eyes) vs abnormal vision (15 eyes) in OPG patients OR OPG (33/64) vs control subjects (20/31)	VA, VF	RNFL	Abnormal vision (15/33 OPG patients): 1 eye abnormal VA only, 7 eyes both abnormal VA and VF	Abnormal vision (15/33 OPG patients): 7 eyes abnormal VF only, 7 eyes abnormal VF and VA
1 participant was imaged using 6x6 mm 300 A-scans per 300 B-scans.														
Avery (2015) [13]	HH-OCT (Bioptigen) or SD-OCT (Spectralis, Heidelberg Engineering)	HH-OCT: 6x6x2 mm volume scan 300 A-scans across 300 B-scans (2.5 s acquisition time) or 1000 A-scans across 100 B-scans (2.8 s acquisition time).	Stable vision (39/45) vs new vision loss (7/10)	VA, VF	RNFL	NA	NA
		Table-top OCT: TruTrack eye tracking (3.5 mm circle over the optic nerve head). Scans were acquired with high-speed mode (768 A-scans) with an automatic real-time setting of 16.					
Fard (2013) [12]	SD-OCT (Spectralis, Heidelberg Engineering)	40.000 A scans/second, scan beam with a wavelength of 870 nm.	Stable vision (18/30) vs vision loss/radiological tumour progression (5/8)	BCVA, VF	RNFL, PPR	Stable vision: 0.52±0.38 logMAR (*n* = 20); vision loss or radiological tumour progression: 0.53±0.18 logMAR (*n* = 6 eyes); *p* = 0.96	Stable vision, MD: -8.4±2.5 dB (*n* = 11); vision loss or radiological tumour progression, MD: -7.75±2.2 dB (*n* = 4 eyes); *p* = 0.6
Gu (2014) [36]	HH-OCT (Bioptigen)	6x6 mm image using 1000 A-scans per 100 B-scans.	Normal vision (19/31) vs abnormal vision (11/16)	VA, VF	RNFL, GCL-IPL	Normal VA/normal VF: 31/31 eyes; abnormal VA/normal VF: 3/16 eyes; normal VA/abnormal VF 6/16 eyes; abnormal VA/abnormal VF 7/16 eyes	Normal VA/normal VF: 31/31 eyes; abnormal VA/normal VF: 3/16 eyes; normal VA/abnormal VF 6/16 eyes; abnormal VA/
		3 participants were imaged using 300 A-scans per 300 B-scans.					
						Abnormal VA 10/16 eyes	Abnormal VF 7/16 eyes
Parrozzani (2018) [34]	SD-OCT (Spectralis, Heidelberg Engineering)	High speed peripapillary RNFL circle scans (circle scan size, 3.5 mm).	NF1 with OPG and normal VA (43 eyes) vs NF1 with OPG and abnormal VA (23 eyes)	VA	RNFL	0.2±0.3 logMAR (range, 0.0–1.4 logMAR; 05% CI: 0.11–0.26 logMAR). Normal VA: 43 eyes; abnormal VA: 23 eyes.	NA
**First author (year)**	**Baseline RNFL thickness**	**Baseline GCL thickness**	**Follow-up VA**	**Follow-up VF**	**Follow-up RNFL thickness**	**Follow-up GCL thickness**	**Cut off value**
Avery (2014) [35]	Average RNFL: normal vision 125.1±13.9 μm; abnormal vision 75.8±16.8 μm (p<0.001: patients with and without vision loss); control 128.1±11.0 μm (p>0.05: control vs normal vision OPG)	NA	NA	NA	NA	NA	Abnormal VA: VA ≥ 0.2 logMAR below age-based norms.
							Abnormal VF: VF defect in any quadrant.
							Abnormal RNFL thickness: lower fifth and first percentile in the normal-vision OPG group.
Avery (2015) [13]	Global RNFL: stable vision 104.7 ± 30.5 μm; new vision loss 84.7 ± 21.0 μm	NA	NA	NA	Stable vision: 103.3 ± 29.5 μm; new vision loss: 66.5 ± 18.3 μm	NA	VA loss: decline ≥ 0.2 logMAR compared to the baseline visit.
							VF loss: Humphrey 24–2: 3 or more contiguous points reaching significance (P < 0.05) (Humphrey 24–2) or any constriction greater than 10 degrees across a minimum of 3 contiguous 15-degree vectors using the V-4-E or I-4-E isopter (Goldmann kinetic perimetry).
							Change in global RNFL thickness: ≥ 10% compared to baseline visit.
Fard (2013) [12]	Average RNFL: stable vision 59.26±8.54 μm (*n* = 30); vision loss or radiological tumour progression: 53.87±3.68 μm (*n* = 8); *p* = 0.09	NA	Stable vision: 0.54±0.37 logMAR (*n* = 20);	Stable vision:	Stable vision: 57.46±8.59 μm (*n* = 30); vision loss or radiological tumour progression; 45.25±3.45 μm (*n* = 8); *p* = 0.0004	NA	VA loss: ≥ 0.2 logMAR compared to baseline visit.
			vision loss or radiological tumour progression: 1.13±0.2 logMAR (*n* = 6); *p* = 0.001	MD: -8.86±2.34 dB (*n* = 11);			VF loss: mean VF mean deviation worsened ≥5 dB.
							Change in average RNFL thickness > 5μm in eyes with progressing OPG.
				vision loss or radiological tumour progression: MD: -14.00±2.1 dB (*n* = 4); *p* = 0.002			
Gu (2014) [36]	Average RNFL: normal vision: outer 4.5 mm, 34.3±5.3 μm; inner 3.0 mm, 27.7±4.1 μm; center 1.5mm, 13.5±2.1 μm. Average RNFL: abnormal vision: outer 4.5 mm, 18.1±7.2 μm; inner 3.0 mm, 16.7±5.3; center 1.5mm, 9.9±2.2 μm.	Average GCL-IPL: normal vision: outer 4.5 mm, 71.6±7.9 μm; inner 3.0 mm, 92.1±9.1 μm; center 1.5mm, 74.3±9.0 μm.	NA	NA	NA	NA	Abnormal VA: VA of ≥ 0.2 logMAR below age-based norms.
							Abnormal VF: VF defect in any quadrant.
							Normal vision: normal VA and VF
							Abnormal vision: abnormal VA and or VF.
		Abnormal vision: outer 4.5 mm, 52.2±5.0 μm; inner 3.0 mm, 60.0±7.9 μm; center 1.5mm, 44.6±12.8 μm					Abnormal RNFL and GCL-IPL: lower fifth and first percentile in the normal-vision group.
Parrozzani (2018) [34]	Global RNFL: 78.7±23.3 μm Normal VA: 88.14±22.6 μm vs Abnormal VA: 61.07±11.6 μm (*p* = 0.0001)	NA	NA	NA	NA	NA	Abnormal VA: any VA below age-based norms.

AUC: area under the curve; BCVA: best corrected visual acuity; CI: confidence interval; GCL-IPL: ganglion cell-inner plexiform layer; HH-OCT: Handheld Spectral Domain Coherence Tomography; MD: mean deviation; NA: not applicable; NF1: Neurofibromatosis type 1; OCT: optical coherence tomography; OPG: optic pathway glioma; PPR: posterior pole retina; RNFL: retinal nerve fiber layer; SD: standard deviation; SD-OCT: Spectral Domain Optical Coherence Tomography; VA: visual acuity; VF: visual field

### Methodological quality of included studies

**Fig 2:**
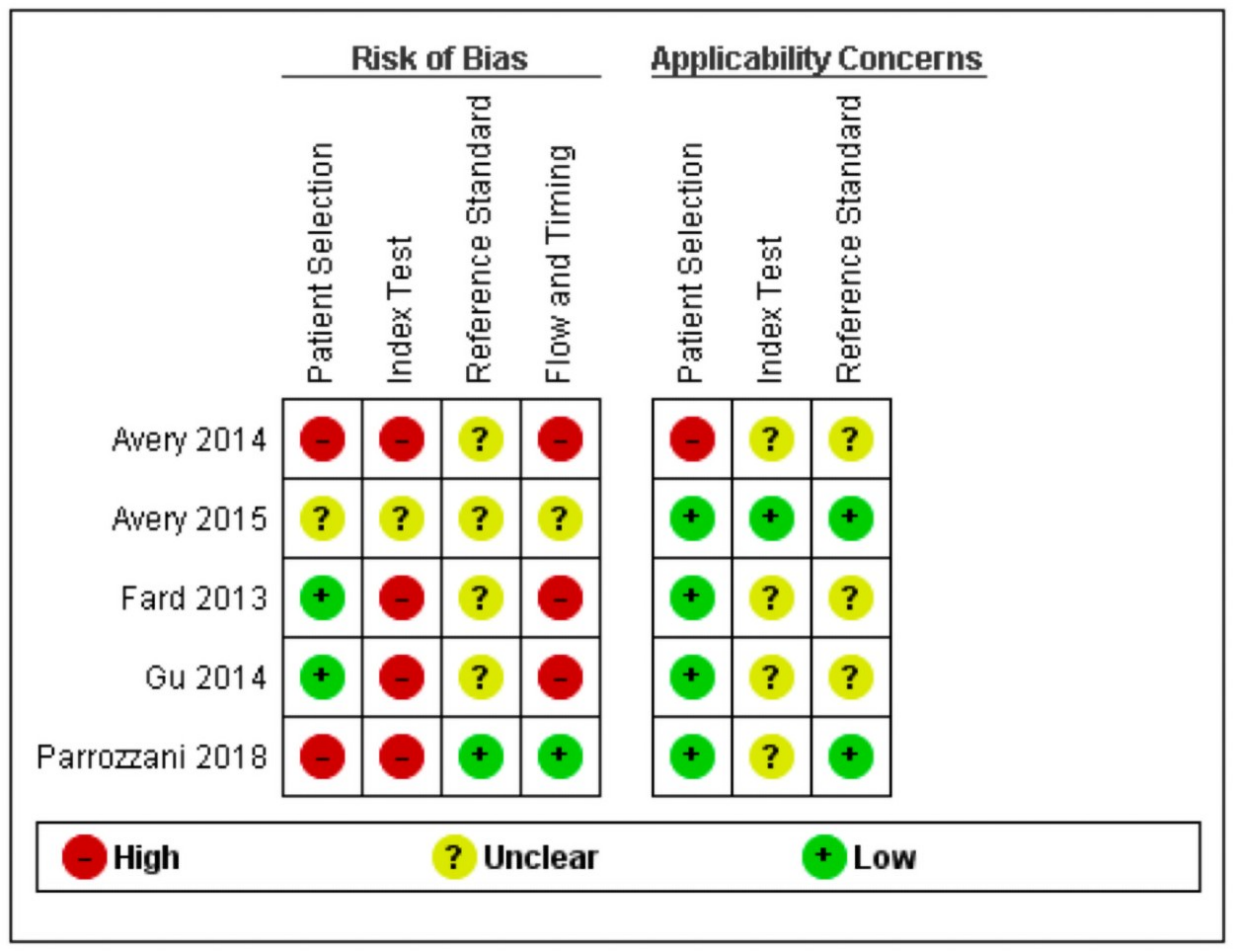
Risk of bias and applicability concerns summary: Review authors’ judgements about each domain for each included study.

**Fig 3:**
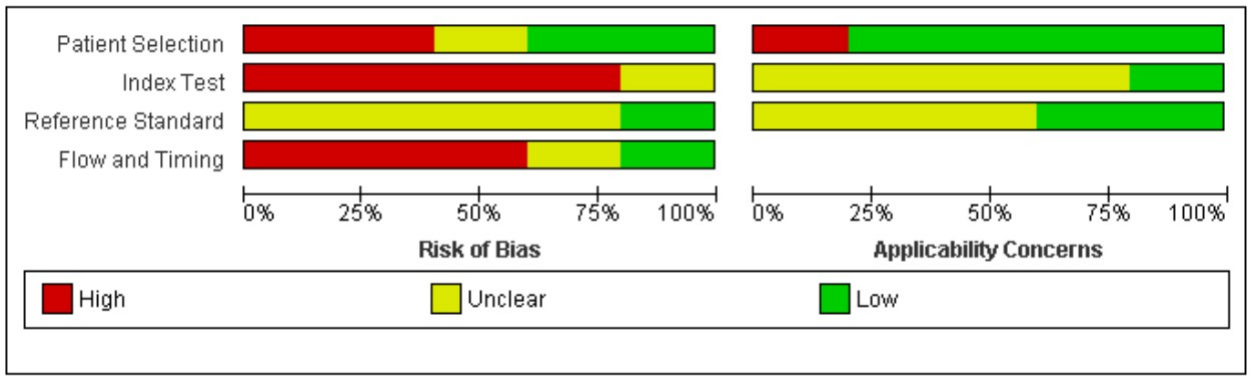
Risk of bias and applicability concerns summary: Review authors’ judgements about each domain for each included study.

The risk of bias of the included diagnostic studies was assessed by the QUADAS-2 tool and the results are shown in Figs 2 and 3. We were not able to apply the QUIPS tool for the risk of bias assessment, because we could not include any prognostic studies in this review. Of the five studies included, one study was judged to have a low or unclear risk of bias for all domains [13]. The other studies all had a high risk of bias for at least two of the four domains assessed with the QUADAS-2 tool [12, 34–36]. Two studies had a high risk of bias for the patient selection domain, due to their case-control design and inappropriate reasons for exclusion of patients [34, 35]. Four studies were judged to have a high risk of bias regarding the index test, because the authors did not specify thresholds for the index test in advance [12, 34–36]. For the reference standard domain, no studies had a high risk of bias. Three studies had a high risk of bias for flow and timing, because not all patients received the same reference standard or not all patients were included in the final analysis [12, 35, 36]. Applicability concerns were rated low or unclear for four studies [12, 13, 34, 36]. One study was judged to have high applicability concerns for patient selection, because also patients without a brain tumour were included (serving as controls) [35].

### Diagnostic accuracy of retinal OCT

One study used VA as reference standard [34], four studies used a combination of both VA and VF as reference standard [12, 13, 35]. Four studies used the RNFL thickness as index test [12, 13, 34, 35] and one study used both the RNFL thickness and GCL-IPL thickness as index tests [36] (Tables 2 and 3). Using criteria for abnormality as less than 5% and less than 1%, Avery et al. (2014) reported an AUC for the average RNFL thickness of 0.96 and 0.97, respectively. Estimates of diagnostic accuracy were highest in patients with RNFL thickness in two or more anatomic quadrants meeting less than 5% (SN = 93.3%; SP = 97.9%; PPV = 93.3%; and NPV = 97.9%) and less than 1% (SN = 93.3%; SP = 100%; PPV = 100%; and NPV = 98.0%) criteria [35]. In the study of Avery et al. (2015) they reported the highest diagnostic accuracy estimates for global average RNFL thickness (SN = 60%; SP = 100%; PPV = 100%; and NPV = 92%) and the inferior quadrant RNFL thickness (SN = 50%; SP = 100%; PPV = 100%; and NPV = 90%) using a threshold of ≥ 10% decline in RNFL thickness. Sensitivity, SP, PPV and NPV for vision loss when 2 or more anatomic quadrants were affected was 70%, 100%, 100% and 94%, respectively [13]. Fard et al. (2013) reported an AUC of 0.94 for the average RNFL thickness. Using a decline of more than 5 μm in RNFL thickness, SN and SP was 100% and 90%, respectively [12]. Using a threshold of less than 5%, Gu et al. (2014) reported an AUC of 0.98 for the GCL-IPL inner macula quadrants and an AUC of 0.94 for the RNFL inner quadrants with PPV of 88.9% and 93.8% and NPV of 100% and 96.8%, respectively [36]. Finally, Parrozzani et al. (2018) reported a best balanced cut-off value of the global RNFL thickness of 76.25 μm (SN = 91%; SP = 76%; PPV = 67%; and NPV = 94%) with an AUC of 0.86. Considering the best balanced cut-off values, estimates of diagnostic accuracy were highest in the superior and inferior RNFL quadrants (superior: SN = 87.0%; SP = 81.4%; PPV = 71.4%; and NPV = 92.1%; inferior: SN = 87.0%; SP = 79.1%; PPV = 69.0%; and NPV = 91.9%) [34].

**Table 3:** Sensitivity, specificity, positive predictive value, negative predictive value and area under the curve of RNFL and GCL-IPL thickness measurements reported in included studies.

First author (year)	SN (%)	SP (%)	PPV (%)	NPV (%)	AUC (95% CI)
Avery (2014) [35]	*RNFL thickness*	*RNFL thickness*	*RNFL thickness*	*RNFL thickness*	*RNFL thickness*
	All quadrants, <5%, <1%: 93.3, 93.3	All quadrants, <5%, <1%: 81.6, 95.9	All quadrants, <5%, <1%: 60.8, 87.5	All quadrants, <5%, <1%: 97.5, 97.9	All quadrants, <5%, <1%: 0.96, 0.97
	Superior, <5%, <1%: 86.7, 73.3	Superior, <5%, <1%: 95.9, 97.9	Superior, <5%, <1%: 86.7, 91.6	Superior, <5%, <1%: 95.9, 92.3	Superior, <5%, <1%: 0.91, 0.85
	Nasal, <5%, <1%: 66.7, 60	Nasal, <5%, <1%: 95.9, 100	Nasal, <5%, <1%: 83.3, 100	Nasal, <5%, <1%: 90.3, 90.7	Nasal, <5%, <1%: 0.81, 0.79
	Inferior, <5%, <1%: 86.7, 86.7	Inferior, <5%, <1%: 93.8, 97.9	Inferior, <5%, <1%: 81.2, 92.9	Inferior, <5%, <1%: 95.8, 96.0	Inferior, <5%, <1%: 0.90, 0.92
	Temporal, <5%, <1%: 73.3, 73.3	Temporal, <5%, <1%: 93.8, 100	Temporal, <5%, <1%: 78.5, 100	Temporal, <5%, <1%: 92.0, 92.4	Temporal, <5%, <1%: 0.83, 0.86
Avery (2015) [13]	*RNFL thickness*	*RNFL thickness*	*RNFL thickness*	*RNFL thickness*	NA
	Global: 60	Global: 100	Global: 100	Global: 92	
	Superior: 60	Superior: 93	Superior: 66	Superior: 91	
	Nasal: 60	Nasal: 98	Nasal: 86	Nasal: 92	
	Inferior: 50	Inferior: 100	Inferior: 100	Inferior: 90	
	Temporal: 40	Temporal: 96	Temporal: 67	Temporal: 88	
	≥ 2 anatomic quadrants with ≥ 10% decline: 70	≥ 2 anatomic quadrants with ≥ 10% decline: 100	≥ 2 anatomic quadrants with ≥ 10% decline: 100	≥ 2 anatomic quadrants with ≥ 10% decline: 94	
Fard (2013) [12]	*RNFL thickness*	*RNFL thickness*	NR	NR	*RNFL thickness*
	Decrease average > 5 μm: 100	Decrease average > 5 μm: 90			Average: 0.94
Gu (2014) [36]	NR	NR	*RNFL thickness*	*RNFL thickness*	*RNFL thickness*
			Outer, <5%, <1%: 92.9, 100	Outer, <5%, <1%: 90.9, 83.8	Outer, <5%, <1%: 0.89 (0.76–0.96), 0.81 (0.66–0.90)
			Inner, <5%, <1%: 93.8, 100	Inner, <5%, <1%: 96.8, 86.1	Inner, <5%, <1%: 0.94 (0.82–0.98), 0.84 (0.71–0.93)
			Center, <5%, <1%: 90.9, 100	Center, <5%, <1%: 83.3, 81.6	Center, <5%, <1%: 0.78 (0.64–0.89), 0.78 (0.64–0.89)
			*GCL-IPL thickness*	*GCL-IPL thickness*	*GCL-IPL thickness*
			Outer, <5%, <1%: 88.9, 93.3	Outer, <5%, <1%: 100, 93.8	Outer, <5%, <1%: 0.97 (0.88–0.99), 0.92 (0.82–0.98)
			Inner, <5%, <1%: 88.9, 100	Inner, <5%, <1%: 100, 96.9	Inner, <5%, <1%: 0.98 (0.92–1.0), 0.96 (0.88–0.99)
			Center, <5%, <1%: 93.3, 100	Center, <5%, <1%: 93.8, 91.2	Center, <5%, <1%: 0.91 (0.79–0.97), 0.90 (0.79–0.97)
Parrozzani (2018) [34]	*RNFL thickness*	*RNFL thickness*	*RNFL thickness*	*RNFL thickness*	*RNFL thickness*
	Global, most sensitive 88 μm, best balanced 76 μm: 100.0, 91.3	Global, most sensitive 88 μm, best balanced 76 μm: 55.8, 76.7	Global, most sensitive 88 μm, best balanced 76 μm: 54.8, 67.7	Global, most sensitive 88 μm, best balanced 76 μm: 100.0, 94.3	Global: 0.86
					Temporal: 0.82
		Temporal, most sensitive 59 μm, best balanced 49 μm: 60.5, 76.7	Temporal, most sensitive 59 μm, best balanced 49 μm: 57.5, 66.7	Temporal, most sensitive 59 μm, best balanced 49 μm: 100.0, 91.7	Superior: 0.86
	Temporal, most sensitive 59 μm, best balanced 49 μm: 100.0, 87.0				Nasal: 0.77
		Superior, most sensitive 115 μm, best balanced 95 μm: 41.9, 81.4	Superior, most sensitive 115 μm, best balanced 95 μm: 47.9, 71.4	Superior, most sensitive 115 μm, best balanced 95 μm: 100.0, 92.1	Inferior: 0.87
	Superior, most sensitive 115 μm, best balanced 95 μm: 100.0, 87.0	Nasal, most sensitive 111 μm, best balanced 54 μm: 2.3, 72.1	Nasal, most sensitive 111 μm, best balanced 54 μm: 35.4, 60.0	Nasal, most sensitive 111 μm, best balanced 54 μm: 100.0, 86.1	
		Inferior, most sensitive 117 μm, best balanced 99 μm: 51.2, 79.1	Inferior, most sensitive 117 μm, best balanced 99 μm: 52.3, 69.0	Inferior, most sensitive 117 μm, best balanced 99 μm: 100.0, 91.9	
	Nasal, most sensitive 111 μm, best balanced 54 μm: 100.0, 78.3				
	Inferior, most sensitive 117 μm, best balanced 99 μm: 100.0, 87.0				

AUC: Area under the curve; CI: Confidence interval; GCL-IPL: Ganglion cell-inner plexiform layer; NPV: Negative predictive value; PPV: Positive predictive value; RNFL: Retinal nerve fiber layer; SN: Sensitivity; SP: Specificity.

## Discussion

We systematically reviewed the diagnostic accuracy and prognostic value of retinal OCT to detect and monitor VA and VF loss in children with a brain tumour. Studies with VA and or VF as reference standard and retinal OCT parameters (RNFL thickness and GCL-IPL thickness) as index test were included in our review. Based on the five included diagnostic studies, we found sensitivity and specificity for average RNFL thickness measurement in children with OPG, ranging from 60.0 to 100.0% and 76.6 to 100%, respectively. Area under the curve for GCL-IPL thickness measurement ranged from 0.91 to 0.98 for centre and inner location, respectively. These findings are in line with the results of a recent review by Banc and associates, reporting that retinal OCT may be a useful instrument in the screening and follow-up of children with OPG [15]. However, the review by Banc and associates did not report on diagnostic accuracy estimates or predictive outcome measures, nor did they evaluate the possible risk of bias of the included studies.

Although our search strategy was designed to identify both diagnostic and prognostic studies on the value of OCT as tool for the detection of VA or VF loss, no prognostic studies were found. To comprehend the relationship between structural retinal changes and functional visual decline in children with a brain tumour, prognostic studies are needed. Understanding this relationship is essential for the use of OCT in addition to standard ophthalmological examination. In adult patients with a brain tumour, several studies support the use of OCT in the early detection and monitoring of VA and VF loss due to chiasmal compression by different types of brain tumours, such as pituitary adenoma, craniopharyngioma and meningioma [37–40]. However, some of these studies also mentioned that functional deficits (e.g. VF defects) from acute or rapidly progressive visual pathway compression typically occur before structural damage on OCT can be established [37–40].

Performing an accurate and reliable ophthalmological examination is imperative as visual decline represents an indication of disease progression and needs consideration for further treatment in children with a brain tumour located along the visual pathway. Unfortunately, VA and VF assessment is often challenging in young children because of limitations in cooperation and concentration; especially in part of the children with NF1-related OPG due to associated cognitive and behavioural problems [41]. Retinal OCT is currently already widely applied for the detection and monitoring of various (ocular) conditions affecting the visual pathway. Due to the high imaging speed of modern spectral-domain table-top and handheld OCT devices, this examination can also be performed in young children with limited cooperation [42]. In addition, recent studies showed adequate repeatability and reproducibility indices for the use of a table-top and handheld OCT devices by a well-trained investigator in these patients [43–47].

Although the use of retinal OCT in children with OPG seems relevant and promising, there are some aspects which need to be considered. One aspect is that normative reference values for RNFL and macular thicknesses for the young population are not incorporated in present-day OCT devices. This means that the results of OCT examination are not automatically compared with values of normal age-matched individuals, as is the case in the adult population. For the interpretation of the OCT results, one needs existing normative databases for the paediatric population [48] or compare consecutive OCT examinations. Another aspect to consider is the possible necessity to perform handheld OCT under general anaesthesia in young children. Three of five authors of studies included in this review reported on the use of handheld OCT in children, with two studies performing handheld OCT in children under general anaesthesia. A disadvantage of incorporating this technique in regular ophthalmological care for young children is that the HH-OCT device is not widely available in (neuro)ophthalmic departments and the application of the device requires specific training and expertise of the operator. Lastly, purchasing the device is expensive, making it less suitable for developing countries.

A possible confounding factor for the use of retinal thickness measurements in children with a brain tumour is the presence of increased intracranial pressure. More than half of the children with a brain tumour present with signs and symptoms of increased intracranial pressure [49]. Eleftheriou and associates found a significantly reduced GCL thickness in adults with normal pressure hydrocephalus compared to healthy individuals (71 μm vs. 79.5 μm, *P* = .001) [50]. Another study by Swanson and associates investigated the potential of OCT to detect increased intracranial pressure in children. In their study, intracranial pressure was correlated with maximal RNFL thickness (r = 0.60, *P* ≤ .001), maximal retinal thickness (r = 0.53, *P* ≤ .001) and maximal anterior retinal projection (r = 0.53, *P* = .003) [51]. The severity and duration of increased intracranial pressure might affect retinal OCT results. Therefore, studies with more precise and earlier assessment of the retinal layers with OCT in larger groups of children with increased intracranial pressure are needed to gain further insight into the relationship between the intracranial pressure, retinal layers and visual function.

The findings of this review should be interpreted in light of several limitations. First, all included studies investigated the diagnostic accuracy of retinal OCT to detect VA or VF loss in children with typical OPG, originating directly from the structures of the optic pathway. Therefore, it is not suitable to extrapolate the results of this review to other types of childhood brain tumours. Secondly, included studies demonstrated considerable heterogeneity in visual function testing methods, scanning protocols and used cut off values for the visual outcomes in the included studies. Not all studies evaluating OCT in children with a brain tumour routinely acquired and or described baseline and follow-up values of VA, VF and OCT parameters. Although we contacted authors of the included studies, additional data was not always provided. Therefore, we had to exclude a number of studies because we had insufficient data to calculate sensitivity and specificity. Thirdly, most of the included studies had a high risk of bias. The main issues found in assessing the risk of bias were regarding the index test and flow and timing domains, i.e. by not using prespecified thresholds for the index test, using different reference standards for patients without mentioning this in the method section and or loss to follow-up of patients. Besides, the studies had relatively low sample sizes, which may lower the methodological quality of included studies. Moreover, inconsistency of the reported data prevented us from pooling all results in a meta-analysis. The included cross-sectional and longitudinal studies provided some insights into the relationship between structural changes and functional visual decline in paediatric patients with OPG. However, investigating this relationship in adequately powered studies including children with other types of brain tumours besides OPG with and without increased intracranial pressure, is highly needed to provide consistent data regarding retinal OCT and to introduce OCT as objective imaging device for the evaluation of the visual status of children with a brain tumour at diagnosis as well as during follow up.

## Conclusion

The literature regarding the diagnostic accuracy and prognostic value of retinal OCT parameters to detect VA or VF loss in children with a brain tumour is scarce. The reviewed literature reveals a relatively high risk of bias. Therefore, we cannot draw any solid conclusions regarding the diagnostic nor the prognostic abilities of retinal OCT to detect VA or VF loss in children with a brain tumour. Well designed, adequately powered studies with prospective longitudinal ophthalmological follow-up and standardized protocols should determine which role is reserved for retinal OCT in the ophthalmological screening and follow-up of children with a brain tumour.

## Supporting information

S1 File Search strategies for electronic databases. (DOCX)

S1 Table QUADAS-2 tool. (DOCX)

S2 Table QUIPS tool. (DOCX)

S3 Table Characteristics of excluded studies. (DOCX)

S1 Checklist PRISMA checklist. (DOC)

## Abbreviations

AUC: Area under the curve
CI: Confidence interval
GCC: Ganglion cell complex
GCL-IPL: Ganglion cell layer—inner plexiform layer
HH-OCT: Handheld optical coherence tomography
logMAR: Logarithm of the Minimum Angle of Resolution
MD: Mean deviation
MRI: Magnetic resonance imaging
NA: Not applicable
NF1: Neurofibromatosis type 1
NPV: Negative predictive value
OCT: Optical coherence tomography
OPG: Optic pathway glioma
PPR: Posterior pole retina
PPV: Positive predictive value
PRISMA: Preferred Reporting Items of Systematic Reviews and Meta-Analyses
QUADAS-2: Quality Assessment of Diagnostic Accuracy
RNFL: Retinal nerve fiber layer
SD: Standard deviation
SD-OCT: Spectrail domain optical coherence tomography
SN: Sensitivity
SP: Specificity
USA: United States of America
VA: Visual acuity
VF: Visual field
