# Supplementary material for: The diagnostic accuracy and prognostic value of OCT for the evaluation of the visual function in children with a brain tumour: A systematic review
Source: PLoS One. 2021 Dec 23;16(12):e0261631. doi: 10.1371/journal.pone.0261631 (PMC8699950; doi:10.1371/journal.pone.0261631)
Supplement: S1 File — (DOCX) [file pone.0261631.s001.docx]

**S1 File. Search strategies for electronic databases**

**Search strategy used for PubMed**

(glioblastoma*[tiab] OR glioma*[tiab] OR optic nerve glioma*[tiab] OR optic pathway glioma*[tiab] OR glioblastoma multiforme[tiab] OR astrocytoma*[tiab] OR craniopharyngioma*[tiab] OR germ cell tumor*[tiab] OR germ cell tumour*[tiab] OR pineal tumor*[tiab] OR pineal tumour*[tiab] OR medulloblastoma*[tiab] OR ependymoma*[tiab] OR atypical teratoid rhabdoid tumor*[tiab] OR atypical teratoid rhabdoid tumour*[tiab] OR ATRT[tiab] OR DIPG[tiab] OR diffuse intrinsic pontine glioma*[tiab] OR oligodendrogli*[tiab] OR choroid plexus tumor*[tiab] OR choroid plexus tumour*[tiab] OR choroid plexus papilloma*[tiab] OR choroid plexus carcinoma*[tiab] OR PNET[tiab] OR primitive neuroectodermal tumor*[tiab] OR primitive neuroectodermal tumour*[tiab] OR "Glioma"[Mesh] OR "Glioma, Subependymal"[Mesh] OR "Optic Nerve Glioma"[Mesh] OR "Astrocytoma"[Mesh] OR "Medulloblastoma"[Mesh] OR "Ependymoma"[Mesh] OR "Neoplasms, Germ Cell and Embryonal"[Mesh] OR optic chiasm*[tiab] OR optic compression*[tiab] OR chiasm compression*[tiab] OR chiasmal compression*[tiab] OR optic tract*[tiab] OR visual pathway*[tiab] OR visual tract*[tiab] OR "Optic Chiasm"[Mesh] OR brain tumor*[tiab] OR brain tumour*[tiab] OR brain neoplasm*[tiab] OR "Brain Neoplasms"[Mesh]) AND (optical coherence tomography[tiab] OR optic coherence tomography[tiab] OR OCT[tiab] OR ganglion cell layer-inner plexiform layer[tiab] OR ganglion cell layer[tiab] OR inner plexiform layer [tiab] OR retinal nerve fiber layer[tiab] OR "Tomography, Optical Coherence"[Mesh])

**Search strategy used for Embase**

(('brain'/exp OR brain) AND tumor*:ti,ab,kw OR (('brain'/exp OR brain) AND tumour*:ti,ab,kw) OR (brain AND neoplasm*:ti,ab,kw) OR 'brain tumor'/exp OR optic chiasm*:ti,ab,kw OR optic compression*:ti,ab,kw OR chiasm compression*:ti,ab,kw OR chiasmal compression*:ti,ab,kw OR optic trac*:ti,ab,kw OR visual pathway*:ti,ab,kw OR visual tract*:ti,ab,kw OR 'optic chiasm'/exp OR glioblastoma*:ti,ab,kw OR glioma*:ti,ab,kw OR (optic AND nerve AND glioma*:ti,ab,kw) OR (optic AND pathway AND glioma*:ti,ab,kw) OR (glioblastoma AND multiforme:ti,ab,kw) OR astrocytoma*:ti,ab,kw OR craniopharyngioma*:ti,ab,kw OR (germ AND cell AND tumor*:ti,ab,kw) OR (germ AND cell AND tumour*:ti,ab,kw) OR (pineal AND tumor*:ti,ab,kw) OR (pineal AND tumour*:ti,ab,kw) OR medulloblastoma*:ti,ab,kw OR ependymoma*:ti,ab,kw OR (atypical AND teratoid AND rhabdoid AND tumor*:ti,ab,kw) OR (atypical AND teratoid AND rhabdoid AND tumour*:ti,ab,kw) OR atrt:ti,ab,kw OR dipg:ti,ab,kw OR (diffuse AND intrinsic AND pontine AND glioma*:ti,ab,kw) OR oligodendrogli*:ti,ab,kw OR (choroid AND plexus AND tumor*:ti,ab,kw) OR (choroid AND plexus AND tumour*:ti,ab,kw) OR (choroid AND plexus AND carcinoma*:ti,ab,kw) OR (choroid AND plexus AND papilloma*:ti,ab,kw) OR pnet:ti,ab,kw OR (primitive AND neuroectodermal AND tumor*:ti,ab,kw) OR (primitive AND neuroectodermal AND tumour*:ti,ab,kw) OR 'central nervous system tumor'/exp OR 'glioma'/exp OR 'optic nerve glioma'/exp OR 'astrocytoma'/exp OR 'medulloblastoma'/exp OR 'ependymoma'/exp) AND (('optical'/exp OR optical) AND ('coherence'/exp OR coherence) AND tomography:ti,ab,kw OR (optic AND coherence AND tomography:ti,ab,kw) OR oct:ti,ab,kw OR (ganglion AND cell AND 'layer inner' AND plexiform AND layer:ti,ab,kw) OR (ganglion AND cell AND layer:ti,ab,kw) OR (inner AND plexiform AND layer:ti,ab,kw) OR (retinal AND nerve AND fiber AND layer:ti,ab,kw) OR 'optical coherence tomography'/exp)

**Search strategy used for Cochrane Library**

(glioblastoma OR glioma OR optic pathway glioma OR glioblastoma multiforme OR astrocytoma OR craniopharyngioma OR germ cell tumour OR germ cell tumor OR pineal tumor OR pineal tumour OR medulloblastoma OR ependymoma OR atypical teratoid rhabdoid tumor OR atypical teratoid rhabdoid tumour OR ATRT OR DIPG OR diffuse intrinsic pontine glioma OR oligodendroglioma OR choroid plexus tumor OR choroid plexus tumour OR choroid plexus papilloma OR PNET OR primitive neuroectodermal tumor OR primitive neuroectodermal tumour OR glioma [MeSH] OR optic nerve glioma [MeSH] OR astrocytoma [MeSH] OR medulloblastoma [MeSH] OR ependymoma [MeSH] OR brain neoplasms [MeSH] OR optic chiasm OR optic compression OR chiasm compression OR chiasmal compression OR optic tract OR visual tract OR visual pathway OR optic chiasm [MeSH] OR brain tumour OR brain tumor OR brain neoplasm) AND (optical coherence tomography OR optic coherence tomography OR rnfl OR gcl OR retinal nerve fiber layer OR ganglion cell layer OR inner plexiform layer OR [Tomography, Optical Coherence] OR OCT)
