# Supplementary material for: The diagnostic accuracy and prognostic value of OCT for the evaluation of the visual function in children with a brain tumour: A systematic review
Source: PLoS One. 2021 Dec 23;16(12):e0261631. doi: 10.1371/journal.pone.0261631 (PMC8699950; doi:10.1371/journal.pone.0261631)
Supplement: S1 Table — (DOCX) [file pone.0261631.s002.docx]

**S1 Table. QUADAS-2 checklist**

| 1. Patient selection | |
| --- | --- |
| *Was a consecutive or random sample of patients enrolled?* | YES: if a study explicitly stated that they enrolled all consecutive patients, a random sample of eligible patients or patients within a certain time frame.  NO: if a different selection procedure was used to include patients.  UNCLEAR: if the patient selection procedure was unclear or not reported. |
| *Was a case-control design avoided?* | YES: if a study explicitly stated that all patients were included from the same group.  NO: if a different selection procedure was used to include patients.  UNCLEAR: if the patient selection procedure was unclear or not reported. |
| *Did the study avoid inappropriate exclusions?* | YES: if there was no clear selection for including a high proportion of eligible patients.  NO: if there were inappropriate exclusions for a high proportion of eligible patients.  UNCLEAR: if no exclusion criteria were reported. |
| *Could the selection of patients have introduced bias?* | HIGH: if one or more signalling questions were answered with NO.  LOW: if all signalling questions were answered with YES.  UNCLEAR: in all other instances. |
| *Are there concerns that the included patients do not match the review question?* | YES: if the diagnostic accuracy and or prognostic value of OCT was assessed in a case-control design, or in a highly selected group of patients.  NO: in all other instances.  UNCLEAR: if there was no description of the included patients. |
| 2. Index test |  |
| *Were the index test results interpreted without knowledge of the results of the reference standard?* | YES: if the index test is always conducted and interpreted prior to the results of the reference standard.  NO: if the index test results were interpreted with knowledge of the results of the reference standard.  UNCLEAR: if blinding was unclear or not reported. |
| *If a threshold was used, was it pre-specified?* | YES: if the threshold used was stated in the methods section.  NO: if the threshold used was not defined before gaining study results; and adapted to optimize sensitivity and or specificity of study results.  UNCLEAR: if it was unclear or not reported how the threshold was selected. NA: if no threshold was used. |
| *Could the conduct or interpretation of the index test have introduced bias?* | HIGH: if one or more signalling questions were answered with NO.  LOW: if all signalling questions were answered with YES.  UNCLEAR: in all other instances. |
| *Are there concerns that the index test, its conduct, or interpretation differ from the review question?* | YES: if thresholds were adapted based on study results and or if the index test results were interpreted unblinded from the reference test study results.  NO: if thresholds were prespecified before receiving study results and if authors were blinded for reference tests results when interpreting the index test results.  UNCLEAR: in all other instances. |
| 3. Reference standard |  |
| *Is the reference standard likely to correctly classify the target condition?* | In this systematic review, the reference standard should be VA and/or VF to correctly classify the visual function. |
| Were the reference standard results interpreted without knowledge of the results of the index test? | YES: if the reference standard results were interpreted without knowledge of or obtained before the results of the index test.  NO: if the reference standard results were interpreted with knowledge of the results of the index test.  UNCLEAR: if blinding was unclear or not reported. |
| Could the reference standard, its conduct, or its interpretation have introduced bias? | HIGH: if one or more signalling questions were answered with NO.  LOW: if all signalling questions were answered with YES.  UNCLEAR: in all other instances. |
| Are there concerns that the target condition as defined by the reference standard does not match the review question? | YES: if VA and or VF were not used as the reference standard(s).  NO: if VA and or VF were used as reference standard.  UNCLEAR: if it was unclear or not reported which reference standard was used. |
| 4. Flow and timing |  |
| *Was there an appropriate interval between index test and reference standard?* | YES: if the time interval between the index test and reference standard was ≤ 2 weeks.  NO: if the time interval between the index test and reference standard was more than two weeks.  UNCLEAR: if the time interval between index test and reference standard was not reported or unclear. |
| *Did all patients receive the same reference standard?* | YES: if all patients received the same reference standard, or if different reference standards were used depending on the age of patients if this was specified in the methods section.  NO: if not all patients received the same reference standard without clarifying for this in the methods section.  UNCLEAR: if the reference standards used were not reported for all included patients. |
| *Were all patients included in the analysis?* | YES: if all patients were included in the analyses.  NO: if not all patients who were recruited into the study were included in the analyses.  UNCLEAR: if it was unclear or not reported. |
| *Could the patient flow have introduced bias?* | HIGH: if one or more signalling questions were answered with NO.  LOW: if all signalling questions were answered with YES.  UNCLEAR: in all other instances. |

The QUADAS-2 tool is adapted from <https://www.bristol.ac.uk/media-library/sites/quadas/migrated/documents/quadas2.pdf>. The criteria for risk of bias assessment have been adjusted in line with this review.

OCT: optical coherence tomography; VA: visual acuity; VF: visual field.
