# Supplementary material for: The diagnostic accuracy and prognostic value of OCT for the evaluation of the visual function in children with a brain tumour: A systematic review
Source: PLoS One. 2021 Dec 23;16(12):e0261631. doi: 10.1371/journal.pone.0261631 (PMC8699950; doi:10.1371/journal.pone.0261631)
Supplement: S2 Table — (DOCX) [file pone.0261631.s003.docx]

**S2 Table. QUIPS TOOL**

| ASSESSMENT FOR RISK OF BIAS | |
| --- | --- |
| **1. Study participation** | **Goal: To judge the risk of selection bias (likelihood that relationship between PF and outcome is different for participants and eligible non-participants).** |
| *Source of target population* | The source population is adequately described, including cases and controls. |
| *Method used to identify population* | The sampling frame and recruitment are adequately described, including methods to identify the sample sufficient to limit potential bias. |
| *Recruitment period* | Period of recruitment is adequately described. |
| *Place of recruitment* | Place of recruitment (setting and geographic location) are adequately described. |
| *Inclusion and exclusion criteria* | Inclusion and exclusion criteria are adequately described. |
| *Adequate study participation* | > 90% of eligible patients do participate in the study. |
| *Baseline characteristics* | The baseline study sample is adequately described for tumor type, age, gender and the presence of NF1 or not. |
| *Study participation summary* | The study sample represents the population of interest on key characteristics, sufficient to limit potential bias of the observed relationship between PF and outcome. |
| **2. Study attrition** | **Goal: To judge the risk of attrition bias (likelihood that relationship between PF and outcome are different for completing and non-completing participants).** |
| *Proportion of baseline sample available for analysis* | Response rate (i.e., proportion of study sample completing the study and providing outcome data) is > 90%. |
| *Attempts to collect information on participants who dropped out* | Attempts to collect information on participants who dropped out of the study are described. |
| *Reasons and potential impact of*  *subjects lost to follow-up* | Reasons for loss to follow-up are provided. |
| *Outcome and prognostic factor*  *information on those lost to follow-up* | Participants lost to follow-up are adequately described for tumor type, age, gender and presence of NF1 or not. There are no important differences between these key characteristics and outcomes in participants who completed the study and those who did not. |
| *Study attrition summary* | Loss to follow-up (from baseline sample to study population analyzed) is not associated with key characteristics sufficient to limit potential bias to the observed relationship between PF and outcome. |
| **3. Prognostic factor measurement** | **Goal: To judge the risk of measurement bias related to how PF was measured (differential measurement of PF related to the level of outcome).** |
| *Definition of the PF* | A clear description of the OCT device and protocol is provided. |
| *Valid and reliable measurement of PF* | The type of OCT device and OCT software are adequately described. Continuous OCT measurements or appropriate cut-offs are used for different OCT parameters. |
| *Method and setting of PF measurement* | The OCT device used is the same for all study participants, or different devices are used depending on the age of patients which is specified in the methods section. |
| *Proportion of data on PF available for analysis* | > 90% of the study sample provided data for OCT measurement. |
| *Method used for missing data* | Imputation is used for missing PF data. |
| *PF measurement summary* | PF is adequately measured in study participants to sufficiently limit potential bias. |
| **4. Outcome measurement** | **Goal: To judge the risk of bias related to the measurement of outcome (differential measurement of outcome related to the baseline level of PF).** |
| *Definition of outcome* | Clear cut-offs are defined for loss of visual acuity and visual field. |
| *Valid and reliable measurement of outcome* | The method of visual acuity and visual field assessment used is adequately valid and reliable. |
| *Method and setting of outcome*  *measurement* | The method and setting of visual acuity and visual field assessment is the same for all study participants, or different methods are used depending on the age of patients which is specified in the methods section. |
| *Outcome measurement summary* | Outcome of interest is adequately measured in study participants to sufficiently limit potential bias. |
| **5. Study confounding** | **Goal: To judge the risk of bias due to confounding (i.e. the effect of PF is distorted by another factor that is related to PF and outcome).** |
| *Important confounders measured* | All important confounders, including type of treatment, presence of NF1 or not, tumour location and researcher blinded or not, are measured. |
| *Definition of the confounding factor* | Clear definitions of the important confounders measured are provided (e.g., including frequency and dose chemotherapy or radiation therapy; timing and frequency of surgery). |
| *Valid and reliable measurement of confounders* | Measurement of all important confounders is adequately valid and reliable. |
| *Method and setting of confounding Measurement* | The method and setting of confounding measurement are the same for all study participants. |
| *Method used for missing data* | Imputation is used for missing confounder data. |
| *Appropriate accounting for confounding* | Important potential confounders are accounted for in the study design and analysis. |
| *Study confounding summary* | Important potential confounders are appropriately accounted for, limiting potential bias with respect to the relationship between PF and outcome. |
| **6. Statistical analysis and reporting** | **Goal: To judge the risk of bias related to the statistical analysis and presentation of results.** |
| *Presentation of analytical strategy* | There is sufficient presentation of data to assess the adequacy of the analysis. |
| *Model development strategy* | The strategy for model building is appropriate and is based on a conceptual framework or model. The selected statistical model is adequate for the design of the study. |
| *Reporting of results* | There is no selective reporting of results. |
| *Statistical analysis and reporting Summary* | The statistical analysis is appropriate for the design of the study, limiting potential for presentation of invalid or spurious results. |

The QUIPS tool is adapted from <https://methods.cochrane.org/sites/methods.cochrane.org.prognosis/files/public/uploads/QUIPS%20tool.pdf>.

The criteria for risk of bias assessment have been adjusted in line with this review.

OCT optical coherence tomography.;NF1: neurofibromatosis type 1; PF: prognostic factor
