# Supplementary material for: The diagnostic accuracy and prognostic value of OCT for the evaluation of the visual function in children with a brain tumour: A systematic review
Source: PLoS One. 2021 Dec 23;16(12):e0261631. doi: 10.1371/journal.pone.0261631 (PMC8699950; doi:10.1371/journal.pone.0261631)
Supplement: S3 Table — (DOCX) [file pone.0261631.s004.docx]

**S3 Table. Characteristics of excluded studies**

| Study | Reason for exclusion |
| --- | --- |
| Abed (2015)(1) | Insufficient data for 2x2 table + comparison between PhNR and the presence or absence of OPG |
| Avery (2014)(2) | No VA and VF data |
| Avery (2014)(3) | No VA, VF and OCT parameters data |
| Avery (2016)(4) | No VA, VF and OCT parameters data |
| Bialer (2013)(5) | Insufficient data for 2x2 table |
| Chang (2010)(6) | Insufficient data for 2x2 table + comparison between NF-1 with OPG and NF-1 non-OPG |
| Estrada (2019)(7) | Insufficient data for 2x2 table + comparison between asymmetric nystagmus with OPG and stable gaze with OPG |
| Hepokur (2018)(8) | Insufficient data for 2x2 table + comparison between OPG (sporadic or secondary to NF-1) and NF-1 non-OPG |
| Mediero (2015)(9) | Insufficient data for 2x2 table |
| Parrozzani (2013)(10) | Insufficient data for 2x2 table + comparison between different visual function tests and the presence or absence of OPG |
| Sahinoglu-Keskek (2018)(11) | Insufficient data for 2x2 table |
| Vagge (2020)(12) | Insufficient data for 2x2 table + comparison between different visual function tests and the presence or absence of OPG |
| Zahavi (2018)(13) | Insufficient data for 2x2 table |

OPG: optic pathway glioma; PhNR: photopic negative response; NF-1: neurofibromatosis type 1; VA: visual

acuity; VF: visual field

**References**

1. Abed E, Piccardi M, Rizzo D, Chiaretti A, Ambrosio L, Petroni S, et al. Functional loss of the inner retina in childhood optic gliomas detected by photopic negative response. Investig Ophthalmol Vis Sci. 2015;56(4):2469–74.

2. Avery RA, Cnaan A, Schuman JS, Chen CL, Glaug NC, Packer RJ, et al. Intra- and inter-visit reproducibility of ganglion cell-inner plexiform layer measurements using handheld optical coherence tomography in children with optic pathway gliomas. Am J Ophthalmol. 2014;158(5):916-923.e1.

3. Avery RA, Cnaan A, Schuman JS, Chen CL, Glaug NC, Packer RJ, et al. Reproducibility of circumpapillary retinal nerve fiber layer measurements using handheld optical coherence tomography in sedated children. Am J Ophthalmol. 2014;158(4):780-787.e1.

4. Avery RA, Mansoor A, Idrees R, Trimboli-Heidler C, Ishikawa H, Packer RJ, et al. Optic pathway glioma volume predicts retinal axon degeneration in neurofibromatosis type 1. Neurology. 2016;87(23):2403–7.

5. Bialer OY, Goldenberg-Cohen N, Toledano H, Snir M, Michowiz S. Retinal NFL thinning on OCT correlates with visual field loss in pediatric craniopharyngioma. Can J Ophthalmol. 2013;48(6):494–9.

6. Chang L, El-Dairi MA, Frempong TA, Burner EL, Bhatti MT, Young TL, et al. Optical coherence tomography in the evaluation of neurofibromatosis type-1 subjects with optic pathway gliomas. J AAPOS. 2010;14(6):511–7.

7. Estrada M, Kelly JP, Wright J, Phillips JO, Weiss A. Visual Function, Brain Imaging, and Physiological Factors in Children With Asymmetric Nystagmus due to Chiasmal Gliomas. Pediatr Neurol. 2019;97(2019):30–7.

8. Hepokur M, Sarici AM. Investigation of retinal nerve fiber layer thickness and ganglion cell layer-inner plexiform layer thickness in patients with optic pathway gliomas. Graefe’s Arch Clin Exp Ophthalmol. 2018;256(9):1757–65.

9. Mediero S, Noval S, Bravo-Ljubetic L, Contreras I, Carceller F. Visual Outcomes, Visual Fields, and Optical Coherence Tomography in Paediatric Craniopharyngioma. Neuroophthalmology. 2015 Jun;39(3):132–9.

10. Parrozzani R, Clementi M, Kotsafti O, Miglionico G, Trevisson E, Orlando G, et al. Optical coherence tomography in the diagnosis of optic pathway gliomas. Invest Ophthalmol Vis Sci. 2013;54(13):8112–8.

11. Sahinoglu-Keskek N, Altan-Yaycioglu R, Canan H, Coban-Karatas M, Erbay A, Yazıcı N, et al. Measurements of Retinal Nerve Fiber Thickness and Ganglion Cell Complex in Neurofibromatosis Type 1, with and Without Optic Pathway Gliomas: A Case Series. Curr Eye Res. 2018;43(3):424–7.

12. Vagge A, Camicione P, Pellegrini M, Gatti G, Capris P, Severino M, et al. Role of visual evoked potentials and optical coherence tomography in the screening for optic pathway gliomas in patients with neurofibromatosis type I. Eur J Ophthalmol. 2020;31(2):698–703.

13. Zahavi A, Toledano H, Cohen R, Sella S, Luckman J, Michowiz S, et al. Use of Optical Coherence Tomography to Detect Retinal Nerve Fiber Loss in Children With Optic Pathway Glioma. Front Neurol. 2018;9(December):1102.
